# Assessing Influence Factors on Daily Ammonia and Greenhouse Gas Concentrations from an Open-Sided Cubicle Barn in Hot Mediterranean Climate

**DOI:** 10.3390/ani11051400

**Published:** 2021-05-14

**Authors:** Provvidenza Rita D’Urso, Claudia Arcidiacono, Francesca Valenti, Giovanni Cascone

**Affiliations:** Department of Agriculture, Food and Environment (Di3A), Building and Land Engineering Section, University of Catania, Via Santa Sofia, 100-95123 Catania, Italy; provvidenza.durso@phd.unict.it (P.R.D.); francesca.valenti@unict.it (F.V.); gcascone@unict.it (G.C.)

**Keywords:** gas concentrations, open barn, climatic parameters, cow behavior, barn management, temperature humidity index, cubicle loose housing system

## Abstract

**Simple Summary:**

Gas concentration is a relevant parameter for the estimation of emissions in dairy farms, but few studies have investigated the influence of cow behavior and barn management on gas concentrations in open buildings. In this study, concentrations of ammonia, methane, and carbon dioxide were investigated in an open dairy barn in a hot Mediterranean climate. Since hot climate conditions cause heat stress to the cows, gas concentrations were statistically analyzed to assess whether variation of environmental and animal-related parameters produced significant effects on the level of gas concentrations in the barn environment. In this study, it was statistically proved that daily gas concentrations were influenced by both the effect of micro-climate conditions, connected with the barn typology, and of barn management on the animals. Therefore, the mitigation strategies for the reduction of these gases could be pursued through the improvement of the barn management aimed at modifying cow behavior and through the control of climatic conditions in relation to the building features.

**Abstract:**

Measurement of gas concentrations constitutes basic knowledge for the computation of emissions from livestock buildings. Although it is well known that hot climate conditions increase gas emissions, in the literature the relation between gas concentrations from open barns and animal-related parameters has not been investigated yet. This study aimed at filling this gap by evaluating daily gas concentrations within an open-sided barn in hot Mediterranean climate. The influence of microclimatic parameters (MC) and cow behavior and barn management (CBBM) were evaluated for ammonia (NH_3_), methane (CH_4_), and carbon dioxide (CO_2_) concentrations. Results showed that both MC and CBBM affected concentrations of NH_3_ (*p* < 0.02), CH_4_ (*p* < 0.001), and CO_2_ (*p* < 0.001). Higher values of NH_3_ concentration were detected during the cleaning of the floor by a tractor with scraper, whereas the lowest NH_3_ concentrations were recorded during animal lying behavior. Measured values of CO_2_ and CH_4_ were highly correlated (C = 0.87–0.89) due to the same sources of production (i.e., digestion and respiration). The different management of the cooling systems during the two observation periods reduced significantly CH_4_ concentrations in the barn when the cooling system in the feeding area was switched off. Based on methodological choices due to the specific barn typology, parameters related to animals can provide information on the variation of gas concentrations in the barn environment in hot climate conditions.

## 1. Introduction

Agriculture and livestock farming are known to be activities with a great environmental impact. Among the main gases emitted from dairy farming, methane (CH_4_) and carbon dioxide (CO_2_), produced during enteric fermentation and manure management, have relevant impacts that contribute to global warming [1]. 

Another atmospheric pollutant, though it is not considered a greenhouse gas (GHG), is ammonia (NH_3_), which is emitted during manure management and produces environmental impact, such us eutrophication, soil acidification, and nutrient-N enrichment of ecosystems [2,3,4,5]. 

The evaluation of the application of mitigation strategies and technologies for emission reduction requires a reliable quantification of gas emissions. This quantification is based in turn on gas concentrations and the ventilation rate of livestock buildings. This latter parameter depends on the gas concentration difference between indoor and outdoor when applying the CO_2_ mass balance method [6] for estimating emissions from naturally ventilated (NV) dairy houses. Therefore, the knowledge of the variation of gas concentration in relation to the main parameters is of utmost importance. 

In this field of investigation, the barn structure, the housing system, the barn management, and the climatic conditions are the main influencing factors of emissions [3,4,7,8,9] and specific techniques or their combination can reduce emissions. However, in the literature there is a lack in the investigation on gas concentrations in open structures with partial or whole absence of perimeter walls. These structures are typical in a hot summer Mediterranean climate (Csa in Koppen classification) where the natural ventilation is generally integrated by a cooling system (e.g., fans and sprinklers) to reduce heat stress of the cows [10,11]. Consequently, the indoor microclimatic conditions are both influenced by the outdoor climatic conditions and the management of the barn (e.g., switching on/off of the cooling system, setpoints for climatic parameters, and number of cooling sessions). 

Microclimatic parameters (MC) of the barn represents one of the main factors that affect animal behavior, physiology, and productivity, as well as emissions of gaseous pollutants [12], especially during the warm seasons. Based on the use of the temperature humidity index (THI) to evaluate the risk of heat stress in cows, Hoffmann et al. [13] synthesized knowledge about activity and lying behavior as non-invasive animal-related parameters. In detail, they described recent outcomes in the literature on how heat stress affected the degree of physical activity. In another study, Porto et al. [10] studied the influence of the alternation of different cooling systems on lying, standing, and feeding behavior under heat stress conditions. They found that the management of the two cooling systems affected the analyzed behaviors. 

However, in the literature the influence of animal-related parameters (e.g., THI, cow activity/lying/feeding index) on gas concentrations has not been thoroughly investigated. Attention has been focused mainly on the relation between concentration and ventilation rate or air exchange rate [14,15,16,17], emissions and climatic variables [8,18,19,20,21,22,23], emissions and barn management or animal activity [23,24,25], and barn management and animal behavior [10,26,27,28]. For instance, Saha et al. [21] analyzed the influence of external wind speed and direction on sample point concentrations. They found that the inflow, i.e., speed and direction of the incoming wind, strongly affects the spatial distribution of NH_3_ and CH_4_ concentrations. The distribution of airflow was investigated by Fiedler et al. [14] within an NV dairy barn. A linear correlation (r = −0.7) showed lower CO_2_ concentrations for higher wind speeds during two weeks of measurements. 

On this basis, this study aimed at increasing knowledge on gas concentrations connections with environmental and animal-related parameters. The hypothesis to be proved was the dependence of gas concentration levels on MC and cow behavior and barn management (CBBM) in hot climate conditions. 

The main objectives of this study were to: (1) study gas concentration distribution in the open barn equipped with a cubicle loose housing system; (2) evaluate the effect of climatic parameters on gas concentrations; and (3) assess the relations between non-invasive animal-related parameters and gas concentrations.

## 2. Materials and Methods

### 2.1. Building and Site Description

Measurements were carried out in a dairy barn equipped with a cubicle loose housing system, located in Pettineo/Pozzilli district (37°01′ N, 14°32′ E) in the province of Ragusa (Italy), at an altitude of 234 m a.s.l.

The dairy house building, about 55.50 m long and 20.80 m wide, has three completely open sides, i.e., the SE, NE, and NW sides, without perimeter walls; the SW side is closed by a continuous wall with small openings; in the NE side there is a row of trees; and the roof is symmetric with a central ridge vent. 

**Figure 1:**
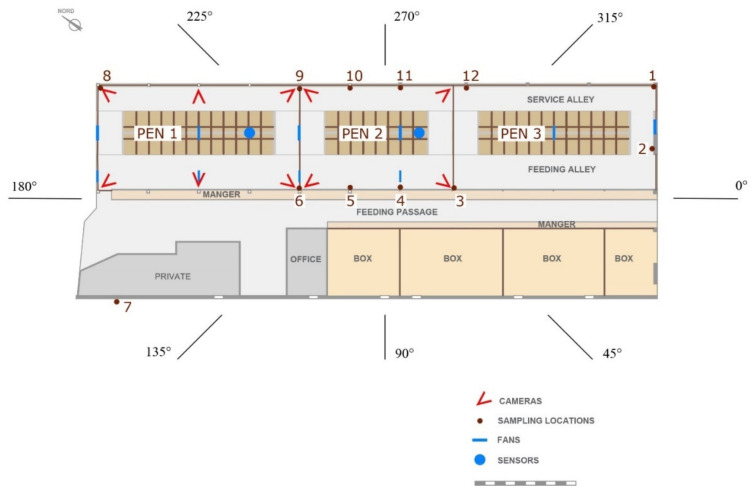
Plan of the barn under study, with location of the axial-flow fans and the monitoring systems. Degree values indicate the direction of airflow in the reference system of the anemometers.

**Figure 2:**
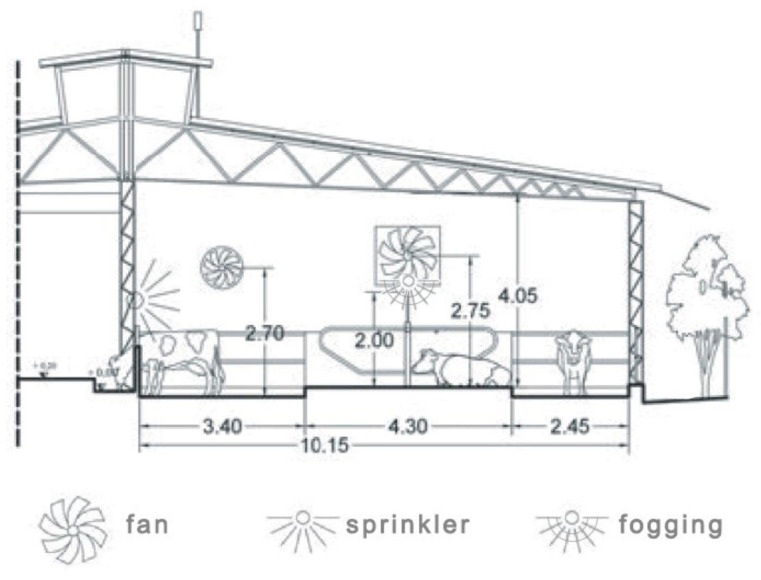
Section of the area under study and location of the cooling system components.

The barn has solid floor and includes three pens for lactating cows, each composed of a resting area, a feeding area, and service alleys (Figure 1). In detail, the resting areas of the three pens are equipped with 64 head-to-head cubicles made of concrete kerbs filled with sand. The building is equipped with two cooling systems (Figure 2): a fogging system with fans located in the resting area; and a sprinkler system with fans located in the feeding alley.

### 2.2. Data Acquisition

Data analyses were carried out in 2016 during spring and summer in two observation periods. These latter were composed of the week from 15/06 to 21/06, named “week 1” (W1) hereafter, and the week from 01/07 to 07/07, named “week 2” (W2) hereafter. In these two weeks, the gas concentrations, climatic and MC, and CBBM were continuously monitored by specific devices and procedures described in the following subsections.

#### 2.2.1. Measurement of Gas Concentrations

Concentrations of CO_2_, NH_3_, and CH_4_ were continuously measured by an INNOVA photo-acoustic analyzer composed of a Multigas Monitor mod 1412 i and a multipoint sampler 1409/12 (Lumasense Technology A/S, Ballerup, Denmark). The sampler system was made of AISI-316 stainless steel and PTFE (polytetrafluoroethylene) tubing to minimize adsorption of samples [29]. The system had 12 inlet channels. An air-filter was attached to the end of each sampling tube to keep the sampler free of particles. The detection limits, declared by the manufacturer, are the following: 0.2 ppm for NH_3_, 0.4 ppm for CH_4_, and 1.5 ppm for CO_2_.

Continuous measurements were carried out at twelve sampling locations (SLs) with a sampling frequency of 15 min. SLs were located within the functional areas of the barn where cow urine and feces are released most, i.e., in the feeding area along the manger and in the service alley at the east side of the barn (Figure 1).

These SLs were located at a height of 20 cm from the barn floor in the animal-occupied zone in order to analyze the gas concentrations where they are most significant for animal presence [30]. The outdoor SL was located at point 7 of Figure 1, at a height of about 3 m above the floor outside the barn and upwind at 2 m from the front of the barn, to acquire background concentrations. The INNOVA system was calibrated by the manufacturer two weeks before the experiment started, and it was operated to acquire data for the experiment.

#### 2.2.2. Climatic and Microclimatic Data Measurements

Measurements of climatic and microclimatic data were carried out by sensors installed inside and outside the barn. Air temperature and relative humidity sensors (Rotronic Italy s.r.l., Milano, Italy) were located in pen 1 and pen 2 at a height of about 2.0 m above the floor and outside the building at the ridge vent above the roof of the barn. The air temperature sensors were platinum thermo-resistances (Pt 100 ohm 0 °C) with a measurement range between −40 and +60 °C and a precision of ±0.2 °C (at 20 °C). The hygrometer was a transducer with a sensitivity of ±0.04%RH/°C and a precision of ±2% (at 20 °C). The position of these two combined sensors was inside a shelter in order to reduce possible inaccuracies due to direct radiation on the sensors. Sensors for the measurement of indoor airflow velocity and direction were located inside the building in pen 2 at a height of about 2.0 m above the floor, and wind speed and direction sensors were placed outside the building at the ridge vent above the roof of the barn. The anemometers were two-dimensional sonic sensors (WindSonic, Gill instruments Ltd., Lymington, UK) characterized by: a velocity measuring interval of 0 ÷ 60 m s^−1^, with a precision of ±2% (at 12 m s^−1^), a resolution of 0.01 m s^−1^, and a threshold of 0.01 m s^−1^; and a direction measuring interval of 0 ÷ 359°, with a precision of ±3% (at 12 m s^−1^), and a resolution of 1°.

The measured values of wind and air temperature and relative humidity, airflow velocity and direction, wind speed and direction, were recorded at intervals of five seconds by a data-logger CR10X (Campbell, City, UK) that every five minutes computed the average values and stored them in memory locations. 

#### 2.2.3. Barn Management and Cow Daily Routine Recordings

Sixty-four Friesian cows were housed in the barn, with primiparous cows mainly located in pen 2 (Figure 1). The daily routine of the cows showed different phases influenced by milking, feed delivery, cleaning, and operation of the cooling systems, which determined cows’ motor activity (e.g., feeding, standing, or walking) or else lying (e.g., resting, ruminating, or sleeping). 

The cleaning was done once a day at about 07:30 a.m. by a mechanical tractor with scraper. In the scraper, a hard rubber was applied to the blade to ensure a better cleaning effect. During the cleaning, the manure was moved to the manure storage area, south of the barn. The milking session was carried out twice a day at about 5:00 a.m. and 5:00 p.m. The feed was delivered every day after cleaning and it was moved into the manger before the first and the second milking sessions. Moreover, cows had ad libitum access to a mixed ratio that was not modified during the two weeks of observation.

Both the cooling systems were manually switched off during the milking sessions and the cleaning of the feeding alley. Fans were automatically switched on when the air temperature exceeded 22 °C, whereas the sprinkler and fogging systems were operated when the air temperature was greater than 27 °C. The forced ventilation was automatically switched off during wetting to avoid the scattering of water. Different operating conditions of the cooling systems were established during data acquisition. Specifically, the sprinkler system in the feeding alley was switched off during W2 in pen 2 in a contextual experiment described by D’Emilio et al. [28].

#### 2.2.4. Behavioral Activity Recordings

Behavioral activity was monitored by a 24-h video-recording system [31], which was composed of ten cameras (Kon.Li.Cor, Perugia, Italy), located at a height of 4 m above the pen floor in the first and in the second pen (Figure 1).

The analysis of cows’ behaviors on the recorded images was carried out by a skilled operator using the scan sampling method [32,33]. The visual assessment was based on the count of the number of cows in activity (e.g., feeding, standing, and walking) and in lying with a frequency of 15 min. These parameters were used in the computation of cow behavioral indices, i.e., cow lying index (CLI), cow standing index (CSI), and cow feeding index (CFI), according to Bava et al. [26]. 

### 2.3. Data Analysis and Statistical Modelling

Data collected during the observation periods were organized in a dataset to carry out statistical analyses on gas concentrations values performed by using Microsoft^®^ Excel and R free software environment.

#### 2.3.1. Gas Concentration Distribution

Variability of gas concentrations at the different sample locations was studied for all the values recorded in the two weeks. Specifically, a one-way analysis of variance (ANOVA) was conducted to assess the gas distribution at different positions of the SLs. Gas concentrations at three groups of SLs were analyzed: central SLs (SL03-SL04-SL05-SL06); perimeter SLs (SL09-SL10-SL11-SL12); corner SLs (SL01-SL02-SL08). On this basis, the groups were separated by Tukey′s honestly significant difference at *p* < 0.05 (post hoc test).

#### 2.3.2. Influence of Micro-Climate Parameters on gas Concentrations

The influence of MC on gas concentrations was analyzed by identifying different ranges of similar climatic conditions. To this aim, wind, and airflow direction data were divided into eight different sectors of 45° (from 0–45° to 315–360°) as it was done in previous studies [14,17]. Figure 1 shows how each sector is related to the orientation of the building. The angles used in the representation gives the direction from which the wind is blowing (e.g., 180° indicates that wind is blowing from 180°). The study of the frequency of data values in those ranges was conducted to obtain the prevailing wind and airflow directions. 

The influence of airflow velocity on gas concentrations was assessed selecting gas concentrations at the prevalent indoor direction. Then, gas concentrations were divided into two groups of indoor airflow velocity (v ≤ 0.5 m/s; v > 0.5 m/s), based on the study carried out by Schrade et al. [19]. Then, for evaluating the equality of their mean values, the one-way ANOVA test was applied.

In the post hoc analyses the mean values were separated by Tukey′s honestly significant difference at *p* < 0.05. 

#### 2.3.3. Influence of Animal-Related Parameters

The influence of animal-related parameters was assessed through two different statistical analyses, based on data grouped by THI and CBBM. 

In the first analyses, the effect of THI on gas concentration expressed the combined effects of temperature and relative humidity on animal stress under specific conditions [10]. The THI was computed by using the following relation [34], suggested by Bohmanova et al. [35] for hot climate conditions and approved by the Italian Ministry for Agricultural, Food and Forestry Policies [36]:

THI = (1.8 × T_db_ + 32) − (0.55 − 0.55 × RH/100) × (1.8 × T_db_ − 26) (1)

where T_db_ is the dry bulb air temperature (°C) and RH is the air relative humidity (%).

The categories of THI for heat stress in dairy cattle were assigned based on the study by Zimbelman and Collier [37] and Hempel et al. [38] and adapted from Armstrong [39] as follows: The first group was related to values of THI < 68 and corresponded to no stress conditions for cows; the second one was related to values 68 ≤ THI ≤ 72 and identified the stress threshold; the third one corresponded to the condition 72 < THI ≤ 78 and described a low risk of thermal stress for cows; and the fourth one was related to the interval 78 < THI < 84 and indicated that cows were in thermal stress. Conditions of emergency for cows (THI ≥ 84) were not recorded in this study. 

In the second statistical analyses, the CBBM conditions were considered as broad categories: They were subdivided into three groups, i.e., cow activity, cow lying, and barn cleaning, and were identified in the video recordings. 

The first group (*cow activity*) included those activities that facilitated the mixing between urea and feces: (1) When cows were moved in groups from the barn to the milking parlour (cow transfer for milking); (2) the permanence of the cows in the feeding alley when they ate (feeding); (3) when cows were in standing position or walked in the alleys (standing/walking). 

The second group (*cow lying*) included the period that cows spent in cubicles resting, rumination, or sleeping. The third group (*barn cleaning*) included the cleaning of the feeding and service alley. In detail, the mixing of urine and feces and their removal, produced by the tractor with a scraper, was considered different from the mixing performed by the cows.

The influence of THI and CBBM on gas concentrations was assessed by using a one-way ANOVA with *p*-value (level of significance) lower than 0.05. If the test was significant (*p* < 0.05), the post hoc test applied was the Tukey test which identified differences between groups. The results related to the two weeks were compared to analyze whether statistical significances were recurrent in both periods. 

Further investigations were carried out to compute the correlation between (i) NH_3_ and CH_4_ and (ii) NH_3_ and CO_2_ and (iii) CO_2_ and CH_4_ following the Pearson correlation coefficient application [21,23]. 

## 3. Results

### 3.1. Gas Concentrations Distribution

**Figure 3:**
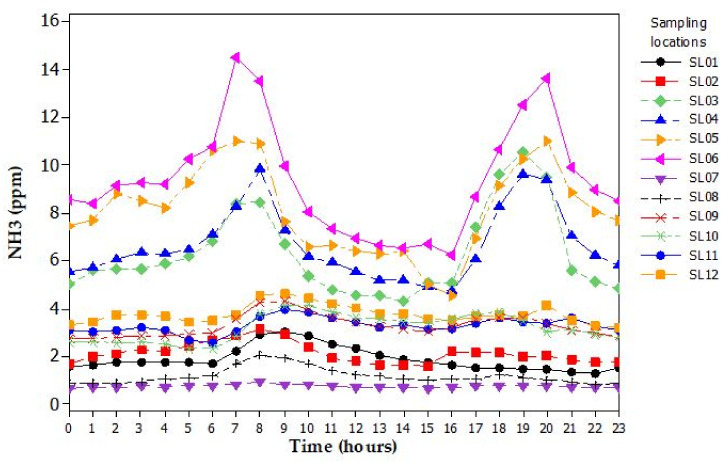
Diurnal variation of ammonia (NH_3_) based on mean values of gas concentrations computed during the observation periods.

Gas concentration profiles for CO_2_, NH_3_, and CH_4_ changed in time and with SLs during the day inside the barn and showed recurrent peaks during each day. Indoor gas concentrations showed a different pattern compared to the outdoor one. Figure 3 shows the variation of NH_3_ concentrations in all SLs during an average day, which was representative of the variation in the two weeks. Gas concentrations were higher in the central SLs than in the perimeter ones. There were two peaks during the day, whereas gas concentrations decreased in the central hours between 9 a.m. and 5 p.m. An example of gas distribution of NH_3_ is described in Figure 3. Gas distribution was uneven inside the barn where the concentration level decreased from the inside to the outside.

A decrease in gas concentration data acquired at the centre of the barn was observed along the longitudinal axis of the building in the NW-SE direction of the airflow. Although symmetrically located in the corner of the barn, NH_3_ concentrations at SL1 were higher than those at SL8. 

The one-way ANOVA highlighted that the four groups of SLs (e.g., central SLs, perimeter SLs, corner SLs, outdoor SL) had a significant difference among them (*p* < 0.001). 

Mean values of gas concentrations, expressed in ppm, with the related standard deviation for each group statistically analyzed for both W1 and W2 are shown in Table 1. 

In both weeks, the central SLs (SL03 to SL06) detected the highest gas concentrations, and they were statistically different from gas concentrations in the other groups of SLs. Results showed a significant difference (*p* < 0.05) in gas concentrations between indoor SLs and the outdoor SL07. On average, it was found that outside concentrations of NH_3_ measured about 18.33% of the indoor concentrations in W1 and 17.21% in W2, i.e., mean indoor NH_3_ concentration was about 5.5 ÷ 6 times the outdoor one. The CO_2_ outdoor concentrations were on average about 82.65% of the indoor concentrations in W1 and 82.30% in W2, i.e., mean indoor CO_2_ concentration was about 1.20 times the outdoor one. Outdoor concentrations of CH_4_ were, on average, about 50.45% of indoor concentrations in W1 and 42.64% in W2, i.e., mean indoor CH_4_ concentration was about 2.00–2.40 times the outdoor one. 

### 3.2. Effect of Climatic Parameters and Micro-Climate Conditions on Gas Concentrations

Statistical measures of climatic parameters in W1 and W2, reported in Table 2, showed no significant differences (*p* < 0.001) for air temperature, relative humidity, and velocity between W1 and W2. Since the prevailing wind and airflow direction moved from NE to SW towards the manure storage area (>85%), W1 and W2 were considered replicates for MC conditions and, thus, the influence of airflow velocity on gas concentrations was assessed.

The group of locations that exhibited the highest values of gas concentrations were selected to perform further analyses and specific datasets were created by filtering them in relation to different MC and CBBM variables. In detail, Table 3 showed the results of the analyses carried out on gas concentrations in relation to the selected indoor airflow velocity ranges in the two weeks. 

The ANOVA showed significant differences for CO_2_, NH_3_, and CH_4_ values of concentrations in air at changing of the airflow velocity. In particular, the results reported in Table 3 show that, when airflow velocity is lower than 0.5 m s^−1^, gas concentration mean values are statistically different from those when airflow velocity is higher than 0.5 m s^−1^. Therefore, gas concentrations are generally high when airflow velocity is low and vice versa. The results of this statistical analysis pointed out that increasing indoor airflow velocity at least above 0.5 m s^−1^ is effective in reducing gas concentration within the breeding environment. 

**Figure 4:**
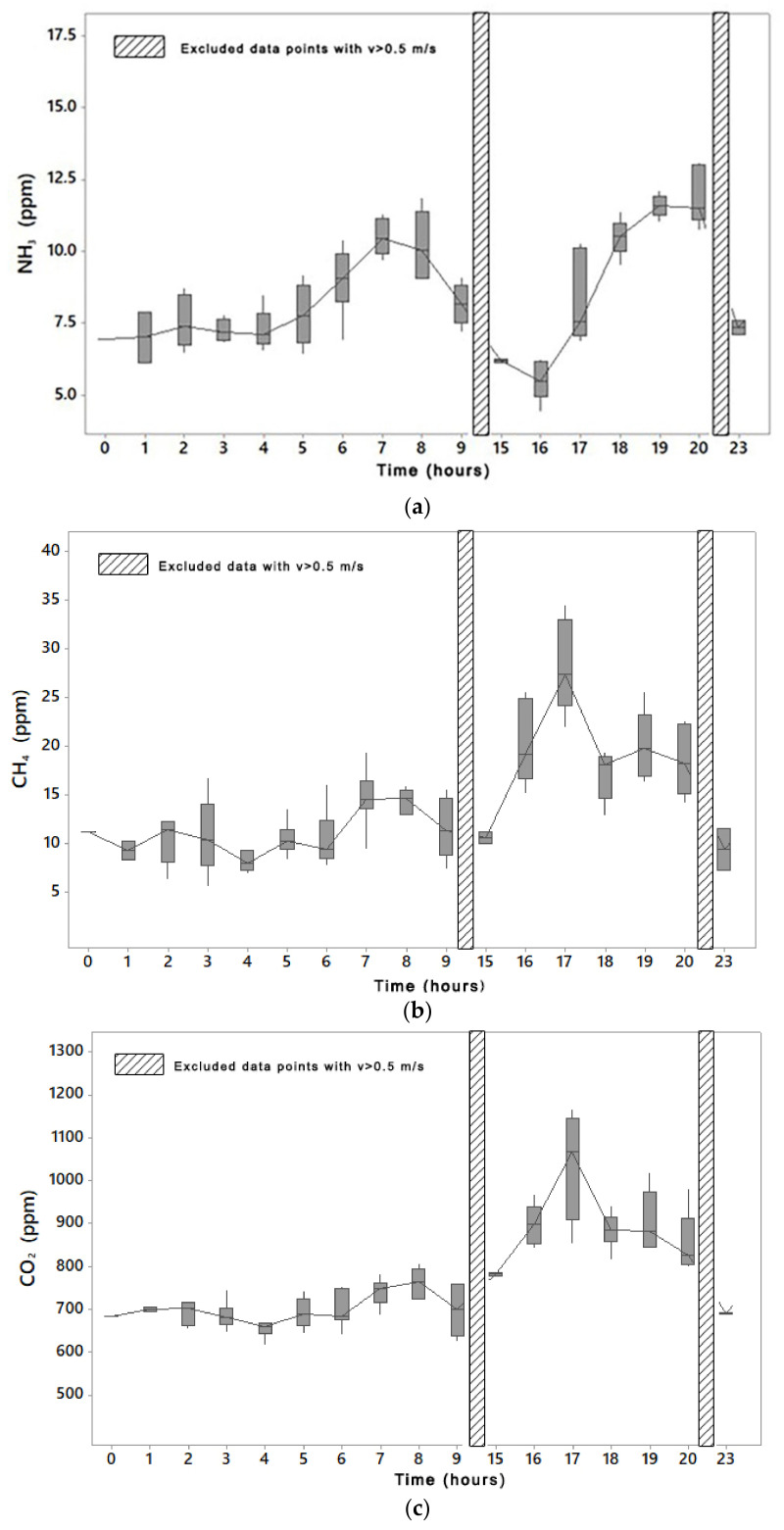
Daily trend of NH_3_ (**a**), CH_4_ (**b**), and CO_2_ (**c**) concentrations, in the central SLs, at low air velocity (v ≤ 0.5 m s^−1^) in W2.

**Figure 5:**
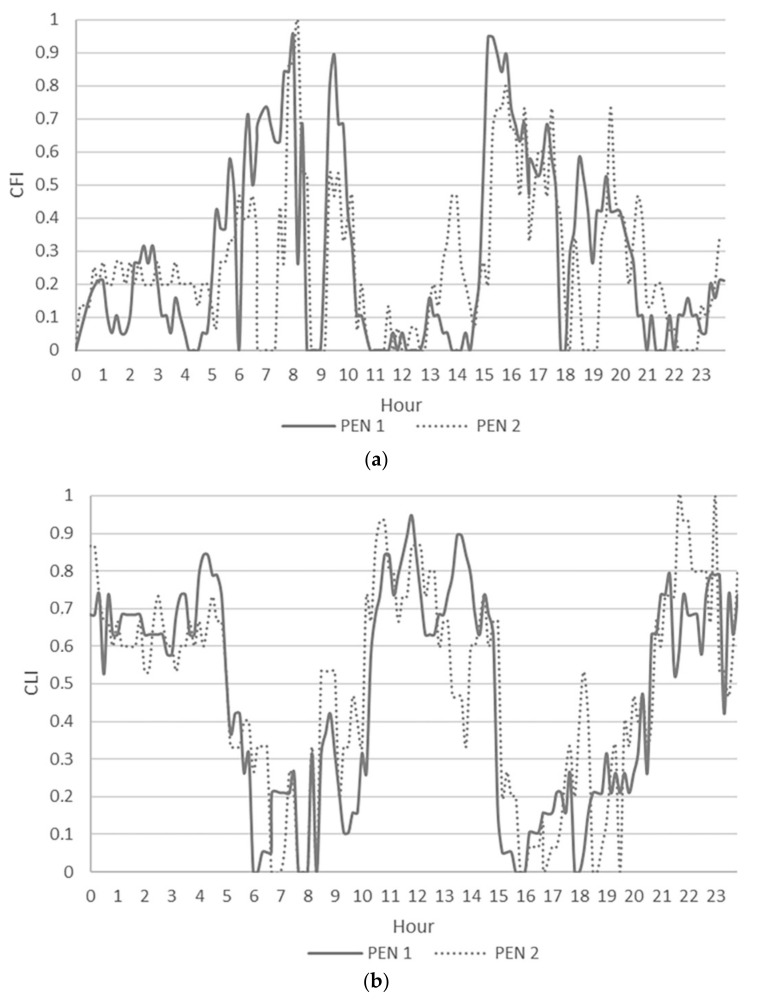
Cow Feeding Index (CFI) (**a**) and Cow Lying Index (CLI) (**b**) during the fifth day of W2, when the sprinkler system in the feeding alley was activated in pen 1 and deactivated in pen 2.

Based on these results, the daily trend of each gas (Figure 4) was analyzed for airflow velocities lower than 0.5 m s^−1^. NH_3_ showed two peaks in both weeks due to cleaning and cows’ feeding activity. The first peak occurred in the morning during cleaning between 7 a.m. and 8 a.m., whereas the second peak occurred at 8 p.m. after about one hour from the end of the milking session, when cows were in feeding. Concerning CO_2_ and CH_4_, the correlation coefficients (C) between these gases were equal to 0.87 in W1 and 0.89 in W2, whereas no correlations were found between NH_3_ and CH_4_ (C = 0.54 in W1 and C = 0.50 in W2) and NH_3_ and CO_2_ (C = 0.41 in W1 and C = 0.43 in W2). The results show that in both weeks the lowest gas concentration values of CO_2_ and CH_4_ were recorded at night when cows were mainly in lying (Figure 5b), whereas higher gas concentrations were found during the afternoon. Moreover, the daily trend of gas concentrations showed that there were two peaks in CH_4_ concentration at 5 p.m. and at 7 p.m. at low airflow velocities. As it was reported in Figure 4, the dataset selection (values related to v ≤ 0.5 m s^−1^) excluded the measurements during two intervals (9 a.m.–3 p.m. and 8 p.m.–10 p.m.) when cows were resting (Figure 5b). In these intervals, gas concentrations always decreased for all gases due to the effect of gas removal by the airflow. With the aim of reducing this effect on data, the groups of gas concentrations at low airflow velocities (v ≤ 0.5 m s^−1^) were analyzed in the following investigations in order to better identify the effect of THI and CBBM on gas concentrations.

### 3.3. The Effect of THI on Gas Concentrations

The analysis of gas concentrations at different THI ranges showed that there was generally a significant relation between THI and gas concentrations in both weeks at an air speed lower than 0.5 m s^−1^. Table 4 reveals that CO_2_ and CH_4_ concentrations were significantly different at the different THI ranges (*p* < 0.001). In detail, the mean values of CO_2_ and CH_4_ were the highest when THI was higher than 78, whereas they were the lowest when THI was under 68. However, CO_2_ and CH_4_ gas concentrations at THI < 68 were not always different from those at 68 ≤ THI ≤ 72, whereas gas concentrations at THI lower than 72 where always different from those at THI higher than 72.

The statistical analyses confirmed that NH_3_ was influenced by THI (*p* < 0.005) in both weeks. The results of the Tukey test post hoc test showed that NH_3_ concentrations when 72 < THI ≤ 78 were significantly different from those when THI ≤ 68. In detail, the highest values of NH_3_ concentrations occurred when 72 < THI ≤ 78. 

### 3.4. Effect of CBBM on Gas Concentrations

During W2, the deactivation of the sprinkler system in the feeding alley of pen 2 changed cow behavior in that area. Figure 5 shows that CFI in pen 2 was generally lower than in pen 1.

The area under the CFI curve in pen 1 was bigger (A = 6.50 CFI day^−1^) than that under the CFI curve in pen 2 (A = 6.17 CFI day^−1^) with a reduction of the time spent at feeding for cows in pen 2. On the contrary, the area under the CLI curve in pen 2 is bigger (A = 11.61 CLI day^−1^) than that under the CLI curve in pen 1 (A = 11.13 CLI day^−1^), increasing time spent in lying. Moreover, a statistical reduction (*p* < 0.006) of CH_4_ concentration was observed from W1 (15 ppm) to W2 (12 ppm).

The application of one-way ANOVA and post hoc test to the gas concentration data acquired during periods at low airflow velocities allowed to statistically prove the influence of CBBM, i.e., activity, lying, and cleaning, on the level of gas concentrations. 

As it was reported in Table 5, results showed a significant influence of CBBM on gas concentrations with *p* < 0.001 in both weeks. Specifically, gas concentrations of CO_2_ and CH_4_ during *cow activity* were always statistically different from those during *cow lying*, whereas NH_3_ concentrations during *barn cleaning*, *cow lying*, and *cow activity* were all significantly different. In detail, gas concentrations during cleaning were the highest and those during lying were the lowest.

## 4. Discussion

### 4.1. Gas Concentration Distribution

Gas distribution of NH_3_, CH_4_, and CO_2_ was uneven inside the barn where the concentration level decreased from inside to outside (Figure 3), similarly to what was observed by Wang et al. [5]. In open structures the effect of boundary conditions produced significantly differences between gas concentrations at central SLs and perimeter or corner SLs. This could be attributed to the absence of the perimeter walls. Moreover, the presence of the feeding alley in the central area of the barn increased the animal activity there. Consequently, the gas concentrations were higher in the central area. The decrease of gas concentrations along the longitudinal axis of the building was to be ascribed to the prevalent airflow direction in the NW-SE direction, composed by the air flux of the fans and the wind direction. In the housing system and the layout of the barn, functional areas influenced the gas concentration distribution. Consequently, multi-location measurements have a relevant role to understand and analyze the heterogeneous distribution of the gas, in agreement with results reported by other authors [17,40,41].

### 4.2. Methodological Considerations

In the barn under study, the environmental conditions related to the activation of axial fans and MC conditions significantly influenced gas concentrations due to the open building structure. 

In order to reduce the effect of gas removal by the airflow on the analyzed data, two main methodological considerations were put forward and the consequent choices were made in this study. The first regarded the selection of gas concentrations in the central SLs and the second involved the selection of gas concentrations at low airflow velocities (v ≤ 0.5 m s^−1^). The observed reduction of gas concentrations from central to perimeter SLs (Table 1) and when operating the cooling systems (Table 3) revealed that airflow velocity increased dilution and flushing. Moreover, the exchange of ventilation air in the building removed polluted air from the barn due to the influence of air movement as it was found by Angrecka and Herbut [9] in a NV dairy barn with open curtains during summer. In fact, the performance of ventilation systems was affected by the power and direction of wind in these open structures [42]. Therefore, these methodological choices allowed us to assess the influence of animal-related parameters on gas concentrations when the environmental parameters had the minimum effect.

### 4.3. Effect of THI on Gas Concentrations

The highest values of CH_4_ and CO_2_ production occurred mainly during the day, when cows were subjected to thermal stress and increased their breathing activity, as it was described by Hoffmann et al. [13]. The statistical analyses (Table 4) delivered the similar results for CO_2_ and CH_4_, in the post hoc tests, due to the strong correlation between CO_2_ and CH_4_. These results are in line with other studies [20,24] and highly depend on the fact that CO_2_ and CH_4_ have the same source of production due to digestion and respiration [43]. Although Zimbelman and Collier [37] found a threshold of THI equal to 68 for cows’ heat stress, in this study the results showed that there was not a significant difference between gas concentrations at THI ≤ 68 and gas concentrations at 68 ≤ THI ≤ 72 for CO_2_ and CH_4_.

The lowest values of gas concentration corresponded to conditions at THI < 68 recorded at night hours when cows are mainly at sleeping. Lower mean values of NH_3_ were found when cows were in thermal stress (78 < THI < 84) because this condition limited their activities as it is further explained in the following Section 4.4. Furthermore, the activation of the sprinkler system during the hottest hours of day (i.e., at high THI values) increased the water on the floor, thus diluting the urines in the puddles, and consequently reducing NH_3_ concentrations in air, similarly to what was described in other studies [4]. 

The highest values of NH_3_ concentrations occurred when 72 < THI ≤ 78 in the period of *cow activity*. The discussion of this point is reported in the following subsection.

### 4.4. Effect of CBBM on Gas Concentrations

During W2, cows in pen 2 reduced time spent at feeding to respond to heat stress. Although there is evidence that cows spend more time standing than lying under stress conditions to increase heat dissipation [13,27], in the barn under study, the activation of the fogging system in the resting area increased cow comfort during the hottest hours of the day and, thus, time spent lying increased [10,11].

Moreover, the presence of sand in the cubicles increased the cooling effect of the fogging system located above the cubicles, as well as ensuring a fresh bed and safe hygienic-sanitary conditions [27,44].

According to other authors [13,26,45], hot climate conditions cause a depressive effect on dry matter intake because the reduction of time spent at feeding reduces the dry matter intake. Moreover, Zetouni et al. [46] proved how there is a high correlation between CH_4_ production and dry matter intake; consequently, the observed decrease in CH_4_ production between W1 and W2 in this study could be ascribed to a reduction of dry matter intake due to hot climate conditions. 

The results of the statistical analyses reported in Table 5 highlighted that barn management strategies (e.g., the operation of cooling systems) are capable of producing an effect on gas concentration levels because cows respond to the management inputs with a different behavior (e.g., cows increased lying when the sprinkler system at the feeding alley was not operated, to maximize their welfare). In detail, the peaks in the daily trend of NH_3_ (Figure 4) are related to cleaning and cow activity. The first peak was the results of the cows’ activity (e.g., feeding and milking) and the consequent cleaning in the morning, whereas the second peak was due to the animal activity (e.g., feeding and milking) in the afternoon at 8 p.m. 

The cleaning interval had the highest concentration of NH_3_ (mean values are 10.70 ppm in W1 and 10.40 ppm in W2) due to both the mixing of urine and feces and the accumulation of NH_3_ during the day [7,30]. In fact, chemical processes for NH_3_ production are triggered by the effects of the mechanical cleaning by the tractor and the urine–feces mixing due to the activity of the cows besides other effects such as the phases of urine excretion. In the barn under study, during the cleaning of feeding and service alleys, cows were moved by the farmer away from the alleys where the tractor was going to operate. Animals were confined in the other part of the pen that was not yet under cleaning, where cows were in activity (e.g., standing, feeding, and walking) and enhanced the mixing of urine and feces on the floor. Therefore, the cleaning operation was the operation with the highest NH_3_ concentrations because it combined the effect of the tractor and the animal activity on the gas production. 

During the second peak of NH_3_, gas concentrations had high values after about one hour from the end of the milking session. This could be explained by the fact that after the milking sessions cows were in feeding. In fact, the farmer switched on the cooling system located in the feeding alley to encourage cows moving towards the feeding area. This action prevented cows from returning to lying immediately after milking; in this case, sand could enter inside the udder sphincter, causing poor hygienic conditions and increasing the risk of mastitis [47]. In the barn under study, after about 30 min from the end of each milking sessions, the sprinkler system in the feeding alley was switched off and the one in the resting area was turned on to make cows leave the feeding area and go to the stalls, in order to encourage cow lying. 

Concerning CO_2_ and CH_4_, findings seem to be in contrast with the main source of gas production, i.e., rumination during lying [48]; however, this is to be attributed to the influence of environmental conditions when cows are often in lying (Figure 5). In the conditions of the barn considered in the experiments, the management of the cooling system modified the trend of these gases by lowering it during the central hours of the day. The daily trend of gas concentrations at low airflow velocities reported in Figure 4 shows that there are two peaks in CH_4_ concentration at 5 p.m. and at 7 p.m. The first peak is seen as being determined by the switching off of the fans, at the beginning of the milking session, which reduced gas dilution. The second peak could be due to the enteric fermentation that occurs approximately 4 h after feeding [48]. Regarding CO_2_, another influencing factor was the passage of the tractor during the scraping of the floor or feed delivering, which contributed to the peaks of gas concentrations in Figure 4c. 

## 5. Conclusions

This study improved knowledge on the influence of environmental and animal-related parameters on gas concentrations in open barns with cubicle housing system. Based on the barn structure, the management of the barn and the response of animals produced effects on the variation of gas concentrations in the barn environment. Knowledge on how gas concentrations are affected by MC and CBBM can be applied to improve the application of mitigations strategies in the daily management of the barn. The control of specific operations (e.g., frequency of manure removal, number of milking, cooling sessions, time of application of urea inhibitors, mechanical efficiency of tractors) could provide precise information to reduce gas concentrations. The analyses performed in this study laid the ground to further study aimed at investigating how the variation of gas concentrations could affect the estimation of emissions in open structures. 

However, this latter point is very challenging for researchers due to the difficulties in the estimation of the ventilation rate by using internal tracer methods (i.e., CO_2_ balance, heat balance, and moisture balance). Further improvements should assess whether it is possible to extend current protocols for measuring gas emissions from NV dairy barn for open structures without the presence of perimeter walls.

## Figures and Tables

**Table 1:** Mean values of gas concentrations (ppm) of carbon dioxide (CO_2_), methane (CH_4_) and ammonia (NH_3_) with related standard deviations (SD) and standard error of mean (SEM) for the sampling location (SL) groups in week 1 (W1) and week 2 (W2). Groups of values that do not share a letter (a, b, c, d) are significantly different within each row.

W1												
Gas	Central SLs			Perimeter SLs			Corner SLs			Outdoor SL		
	Mean	SD	SEM	Mean	SD	SEM	Mean	SD	SEM	Mean	SD	SEM
CO_2_	724 ^a^	124	5	597 ^b^	43	1.7	580 ^b^	33	43	524 ^c^	19	0.7
CH_4_	15 ^a^	6	0.2	8 ^b^	3	0.1	7 ^b^	3	3	5 ^c^	2	0.1
NH_3_	7.4 ^a^	2.4	0.1	3.4 ^b^	0.8	0.03	1.8 ^c^	0.6	0.8	0.8 ^d^	0.1	4 × 10^−3^
**W2**												
**Gas**	**Central SLs**			**Perimeter SLs**			**Corner SLs**			**Outdoor SL**		
	**Mean**	**SD**	**SEM**	**Mean**	**SD**	**SEM**	**Mean**	**SD**	**SEM**	**Mean**	**SD**	**SEM**
CO_2_	741 ^a^	102	4	603 ^b^	33	1	588 ^c^	24	0.9	530 ^d^	12	0.5
CH_4_	12 ^a^	5	0.2	5 ^c^	3	0.1	6 ^b^	2	0.1	4 ^d^	2	0.1
NH_3_	7.8 ^a^	2.0	0.1	3.4 ^b^	0.6	0.02	1.8 ^c^	0.4	0.02	0.8 ^d^	0.2	0.01

**Table 2:** Statistical measures for indoor and outdoor climatic parameters in W1 and W2, and pairwise comparison for each parameter between the two weeks. Groups of values that do not share a letter (a, b) are significantly different within each row.

Climatic Parameter	W1						W2					
	Indoor			Outdoor			Indoor			Outdoor		
	Mean	SD	SEM	Mean	SD	SEM	Mean	SD	SEM	Mean	SD	SEM
Air temperature (°C)	26.6 ^a^	5.9	0.2	25.5 ^a^	5.9	0.2	27.4 ^a^	4.8	0.2	26.4 ^a^	4.5	0.2
Air relative humidity (%)	57.9 ^a^	19.3	0.7	47.3 ^a^	18.7	0.7	55.2 ^a^	16.4	0.6	44.3 ^a^	16.1	0.6
Airflow velocity (ms^−1^)	0.76 ^a^	0.50	0.02	1.95 ^a^	1.55	0.06	0.68 ^a^	0.39	0.02	1.24 ^b^	0.59	0.02

**Table 3:** Results of the statistical analyses for gas concentrations (ppm) of CO_2_, CH_4_ and NH_3_, in the central SLs, at different airflow velocities v (ms^−1^) during W1 and W2. Groups of values that do not share a letter (a, b) are significantly different.

W1 (15.06.16–21.06.16)				W2 (1.07.16–07.07.16)			
Range	Mean	SD	SEM	Range	Mean	SD	SEM
CO_2_				CO_2_			
v ≤ 0.5	743 ^a^	177	10	v ≤ 0.5	778 ^a^	157	9
v > 0.5	708 ^a^	164	9	v > 0.5	705 ^b^	80	5
**CH_4_**				**CH_4_**			
v ≤ 0.5	17 ^a^	10	0.6	v ≤ 0.5	15 ^a^	8	0.4
v > 0.5	13 ^b^	9	0.5	v > 0.5	9 ^b^	4	0.2
**NH_3_**				**NH_3_**			
v ≤ 0.5	8.8 ^a^	2.4	0.1	v ≤ 0.5	8.8 ^a^	2.2	0.1
v > 0.5	6.4 ^b^	2.0	0.1	v > 0.5	6.6 ^b^	1.3	0.1

**Table 4:** Results of statistical analyses for gas concentrations (ppm) of CO_2_, CH_4_ and NH_3_, in the central SLs, at different ranges of temperature-humidity index (THI), for low airflow velocity values (v ≤ 0.5 ms^−1^). Groups of values that do not share a letter (a, c, b, d) are significantly different.

W1 (15.06.16–21.06.16).				W2 (1.07.16–07.07.16)			
Range	Mean	SD	SEM	Range	Mean	SD	SEM
CO_2_				CO_2_			
78 < THI < 84	1141 ^a^	234	33	78 < THI < 84	943 ^a^	169	20
72 < THI ≤ 78	843 ^b^	184	20	72 < THI ≤ 78	854 ^b^	160	16
68 ≤ THI ≤ 72	716 ^c^	81	8	68 ≤ THI ≤ 72	714 ^c^	58	5
THI < 68	656 ^d^	50	5	THI < 68	675 ^c^	29	4
**CH_4_**				**CH_4_**			
78 < THI < 84	35 ^a^	13	2	78 < THI < 84	23 ^a^	10	1
72 < THI ≤ 78	18 ^b^	10	1	72 < THI ≤ 78	18 ^b^	9	0.9
68 ≤ THI ≤ 72	13 ^c^	5	0.5	68 ≤ THI ≤ 72	13 ^c^	5	0.5
THI < 68	12 ^c^	5	0.5	THI < 68	9 ^d^	2	0.3
**NH_3_**				**NH_3_**			
72 < THI ≤ 78	9.3 ^a^	3.1	0.3	72 < THI ≤ 78	10.3 ^a^	2.1	0.2
68 ≤ THI ≤ 72	9.0 ^ab^	1.8	0.2	68 ≤ THI ≤ 72	8.5 ^b^	1.8	0.2
78 < THI < 84	8.3 ^ab^	3.4	0.5	78 < THI < 84	8.2 ^b^	2.4	0.3
THI < 68	8.2 ^b^	1.6	0.2	THI < 68	8 ^b^	1	0.1

**Table 5:** Results of statistical analyses on gas concentrations (ppm), in the central SLs, at different cow behaviour and barn management (CBBM) in the weeks considered, during periods at low airflow velocities (v ≤ 0.5 ms^−1^). Groups of values (a, b, c) that do not share a letter are significantly different.

W1 (15.06.16–21.06.16)				W2 (1.07.16–07.07.16)			
CBBM	Mean	SD	SEM	CBBM	Mean Value	SD	SEM
CO_2_				CO_2_			
activity	821 ^a^	206	17	activity	852 ^a^	168	12
cleaning	735 ^b^	147	20	cleaning	775 ^b^	138	19
lying	687 ^b^	105	10	lying	690 ^c^	50	5
**CH_4_**				**CH_4_**			
activity	19 ^a^	12	1	activity	18 ^a^	9	0.6
cleaning	15 ^b^	8	1	cleaning	18 ^a^	10	1
lying	12 ^b^	5	0.5	lying	10 ^b^	4	0.4
**NH_3_**				**NH_3_**			
cleaning	10.9 ^a^	1.9	0.3	cleaning	10.6 ^a^	1.4	0.2
activity	8.8 ^b^	2.7	0.2	activity	9.0 ^b^	2.4	0.2
lying	8.1 ^c^	1.6	0.2	lying	7.4 ^c^	0.8	0.1

## Data Availability

Data available on request due to privacy restrictions.
